# A new hydrate of magnesium carbonate, MgCO_3_·6H_2_O

**DOI:** 10.1107/S2053229620001540

**Published:** 2020-02-13

**Authors:** Christine Rincke, Horst Schmidt, Wolfgang Voigt

**Affiliations:** a Institute of Inorganic Chemistry, TU Bergakademie Freiberg, Leipziger Strasse 29, D-09599 Freiberg, Germany

**Keywords:** magnesium, carbonate, low-temperature hydrate, Raman spectroscopy, crystal structure

## Abstract

During investigations of the formation of hydrated magnesium carbonates, a sample of the previously unknown magnesium carbonate hexa­hydrate (MgCO_3_·6H_2_O) was synthesized in an aqueous solution at 273.15 K. The crystal structure consists of edge-linked isolated pairs of Mg(CO_3_)(H_2_O)_4_ octa­hedra and noncoordinating water mol­ecules, and exhibits similarities to NiCO_3_·5.5H_2_O (hellyerite). The recorded X-ray diffraction pattern and the Raman spectra confirmed the formation of a new phase and its transformation to magnesium carbonate trihydrate (MgCO_3_·3H_2_O) at room temperature.

## Abstract 2

The formation of a previously unknown higher hydrated magnesium carbonate, MgCO_3_·6H_2_O, was confirmed. Its crystal structure differs from the other known magnesium carbonates significantly, but it exhibits similarities to NiCO_3_·5.5H_2_O.

## Introduction   

In the MgO–H_2_O–CO_2_ system, besides the thermodynami­cally stable MgCO_3_ (magnesite), a variety of hydrated mag­nesium carbonates are known, which can be divided in basic magnesium carbonates, containing OH^−^ ions [Mg_5_(CO_3_)_4_(OH)_2_·*n*H_2_O and Mg(CO_3_)(OH)_2_·*n*H_2_O], and neutral mag­nesium carbonates with the com­position MgCO_3_·*n*H_2_O (Hopkinson *et al.*, 2012 ▸; Jauffret *et al.*, 2015 ▸). All these phases are of significant relevance in various technological processes, in geological explorations, mineral conversion in the sequestration of CO_2_ and in biomineralization (Chaka & Felmy, 2014 ▸). Nevertheless, there are many open questions with respect to the conditions of formation, the characterization, the crystal structure and the stability of higher hydrated neutral magnesium carbonates (Hänchen *et al.*, 2008 ▸; Hopkinson *et al.*, 2012 ▸; Rincke, 2018 ▸).

The most frequently investigated neutral magnesium carbonate hydrate, MgCO_3_·3H_2_O (mineral name: nesquehonite), can be synthesized in the temperature range between 283.15 and 325.15 K (Giester *et al.*, 2000 ▸; Frost & Palmer, 2011 ▸; Jauffret *et al.*, 2015 ▸; Hänchen *et al.*, 2008 ▸; Gloss, 1937 ▸; Takahashi & Hokoku, 1927 ▸; Hopkinson *et al.*, 2012 ▸).

At lower temperatures, the penta­hydrate, *i.e.* MgCO_3_·5H_2_O (mineral name: lansfordite), is known. Its crystal structure (monoclinic space group *P*2_1_/*m*) was determined by Liu *et al.* (1990 ▸) from a synthetic sample and by Nestola *et al.* (2017 ▸) from a mineral. Several possibilities are described to synthesize lansfordite (Ming & Franklin, 1985 ▸; Liu *et al.*, 1990 ▸). In order to obtain large prismatic crystals, CO_2_ can be bubbled through an aqueous suspension of MgO and, subsequently, the crystallization can be carried out in the filtered solution at low temperature (Liu *et al.*, 1990 ▸). However, the authors (Liu *et al.*, 1990 ▸) did not provide information about the exact CO_2_ pressure, the regime of temperature, the concentration of magnesium ions in the solution and the time needed for crystallization. According to Ming & Franklin (1985 ▸), these factors are important to avoid the formation of nesquehonite. Furthermore, the solubility of lansfordite is highly dependent on temperature and on CO_2_ pressure (Königsberger *et al.*, 1999 ▸; Takahashi & Hokoku, 1927 ▸; Rincke, 2018 ▸). Besides that, there are contradictory statements about the temperature and the rate of conversion of the penta­hydrate to the trihydrate. Some research groups have recorded a transition temperature between 283.15 and 288.15 K (Takahashi & Hokoku, 1927 ▸; Gloss, 1937 ▸; Yanaťeva & Rassonskaya, 1961 ▸; Hill *et al.*, 1982 ▸; Langmuir, 1965 ▸; Ming & Franklin, 1985 ▸), while others observed the stability of synthesized and natural samples of lansfordite at room temperature over a period of a few months (Liu *et al.*, 1990 ▸; Nestola *et al.*, 2017 ▸). Neutral magnesium carbonates with a water content greater than five units per formula are not known up to now. Such highly hydrated neutral carbonates of other bivalent metal ions have been found only for calcium, *i.e.* CaCO_3_·6H_2_O (ikaite; Hesse *et al.*, 1983 ▸), and nickel, *i.e.* NiCO_3_·5.5H_2_O (hellyerite; Bette *et al.*, 2016 ▸). Our own investigations should elucidate the conditions of formation of the magnesium carbonate hydrates.

## Experimental   

### Synthesis and crystallization   

To obtain crystals of MgCO_3_·6H_2_O suitable for single-crystal diffraction analysis (see V11 in Table S1 of the supporting information), carbon dioxide was bubbled through a suspension of magnesium oxide in deionized water (3.1 g, Magnesia M2329, p.a.) for 22 h at 273.15 K. After that, the solution was filtered and stored without stirring at 273.15 K for 16 d until the product crystallized. The product was filtered off for characterization by powder X-ray diffraction and Raman spectroscopy. For intensity data collection, a prismatic crystal of MgCO_3_·6H_2_O was recovered from a droplet of its mother liquor and mounted rapidly in the cold (200 K) stream of nitro­gen gas of the diffractometer.

### Powder X-ray diffraction   

The powder X-ray diffraction (PXRD) patterns were taken for phase identification with a Bruker D8 Discover laboratory powder diffrac­tometer in the Bragg–Brentano set-up (Cu *K*α_1_ radiation, Vantec 1 detector). The samples were prepared as flat plates and measured at low temperatures (about 273.15 K) with a home-made cooling box (Rincke, 2018 ▸).

### Raman spectroscopy   

The Raman spectra were recorded shortly after synthesis with a Bruker RFS100/S FT spectrometer at room temperature (Nd/YAG laser, wavelength of the laser = 1064 nm).

### Refinement   

Crystal data, data collection and structure refinement details are given in Table 1 ▸. The positions of the H atoms could be located from residual electron-density maxima after further refinement and were refined isotropically.

## Results and discussion   

### Conditions of formation and characterization of magnesium carbonate hexa­hydrate   

**Figure 1:**
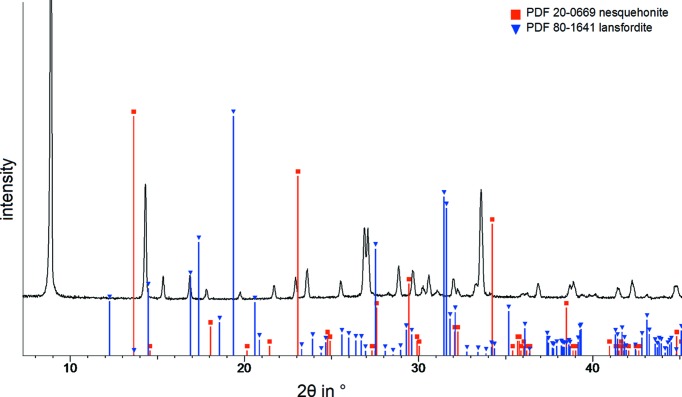
Powder XRD pattern of MgCO_3_·6H_2_O at low temperature (∼273.15 K, Cu *K*α radiation) and reference data for MgCO_3_·3H_2_O (PDF 20-0669) and MgCO_3_·5H_2_O (PDF 80-1641).

On the basis of the information of Liu *et al.* (1990 ▸) for the formation of lansfordite, CO_2_ was bubbled through aqueous MgO suspensions with various concentrations. After filtration of the solution, the product crystallized at low temperature (273.15–278.15 K), while the CO_2_ pressure was decreased by slow degassing of the CO_2_ and the solubility product of the carbonate was exceeded. The detailed experimental conditions are given in the supporting information (see Table S1). Characterization of the product with PXRD revealed that nesquehonite is formed at low MgO concentrations, while an unknown phase crystallizes from the solutions at higher Mg^2+^ concentrations, near the solubility of lansfordite at *p*(CO_2_) = 1 bar [m(Mg^2+^) = 0.386 mol kg^−1^(H_2_O) at 273.15 K] (Königsberger *et al.*, 1999 ▸; Rincke, 2018 ▸). Fig. 1 ▸ shows the PXRD pattern of the new product phase in com­parison with the reference data for MgCO_3_·3H_2_O and MgCO_3_·5H_2_O. The com­position of this unknown phase was determined by single-crystal diffraction as MgCO_3_·6H_2_O. The penta­hydrate was not found in our investigations.

**Figure 2:**
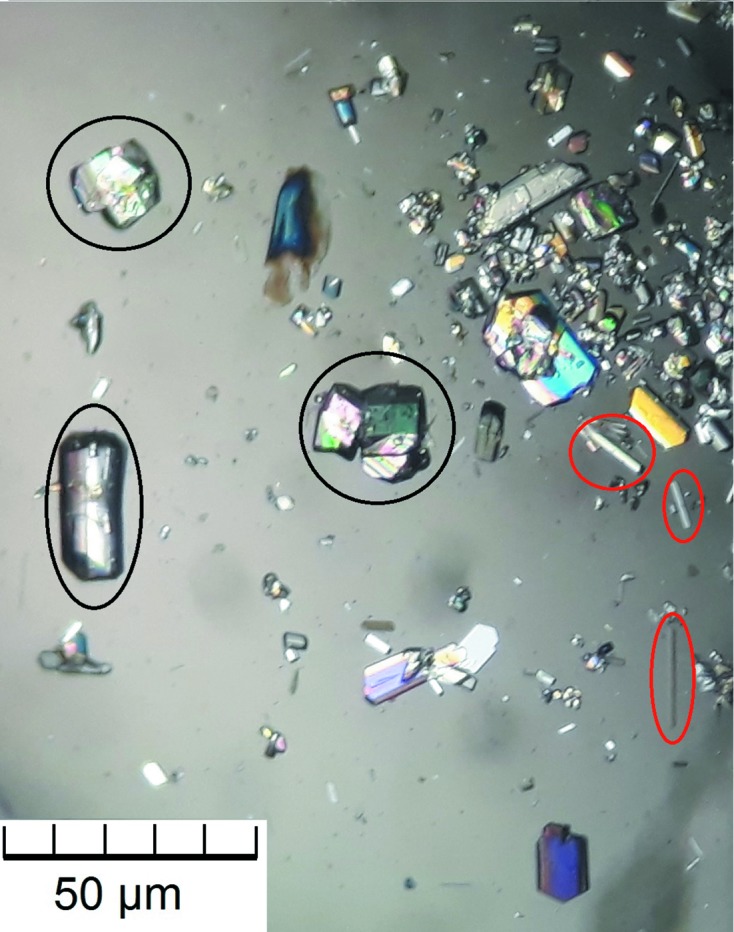
Microscopic image of prismatic MgCO_3_·6H_2_O crystals (framed in black) which are partly inter­grown. The red-framed crystals are the typical needles of the transformation product MgCO_3_·3H_2_O.

**Figure 3:**
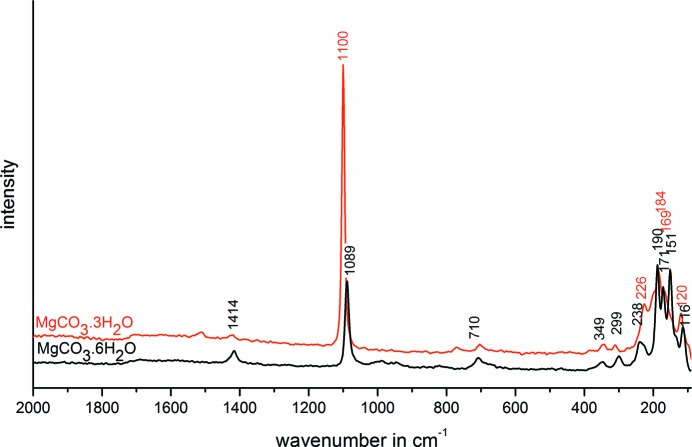
Raman spectrum of MgCO_3_·6H_2_O (black) in com­parison with the transformation product MgCO_3_·3H_2_O (red) after storage at room temperature.

Large prismatic crystals of MgCO_3_·6H_2_O were obtained while using a longer time of crystallization of 16 d (see V11 in Table S1 of the supporting information). These crystals, which are partly inter­grown, convert in a few minutes at room temperature into the typical needles of MgCO_3_·3H_2_O (Fig. 2 ▸). The process of phase transformation could also be seen by means of Raman spectroscopy (Fig. 3 ▸). The assignments of the band positions in com­parison with the spectra of nesquehonite and lansfordite are given in the supporting information (Table S2).

### Crystal structure of magnesium carbonate hexa­hydrate   

Magnesium carbonate hexa­hydrate crystallizes in the ortho­rhom­bic space group *Pbam* (No. 55). The Mg1 atom is located on a twofold axis of symmetry. Atoms C1, O1 and O5, and the water mol­ecule H6*A*—O6—H6*B* are positioned on a mirror plane.

**Figure 4:**
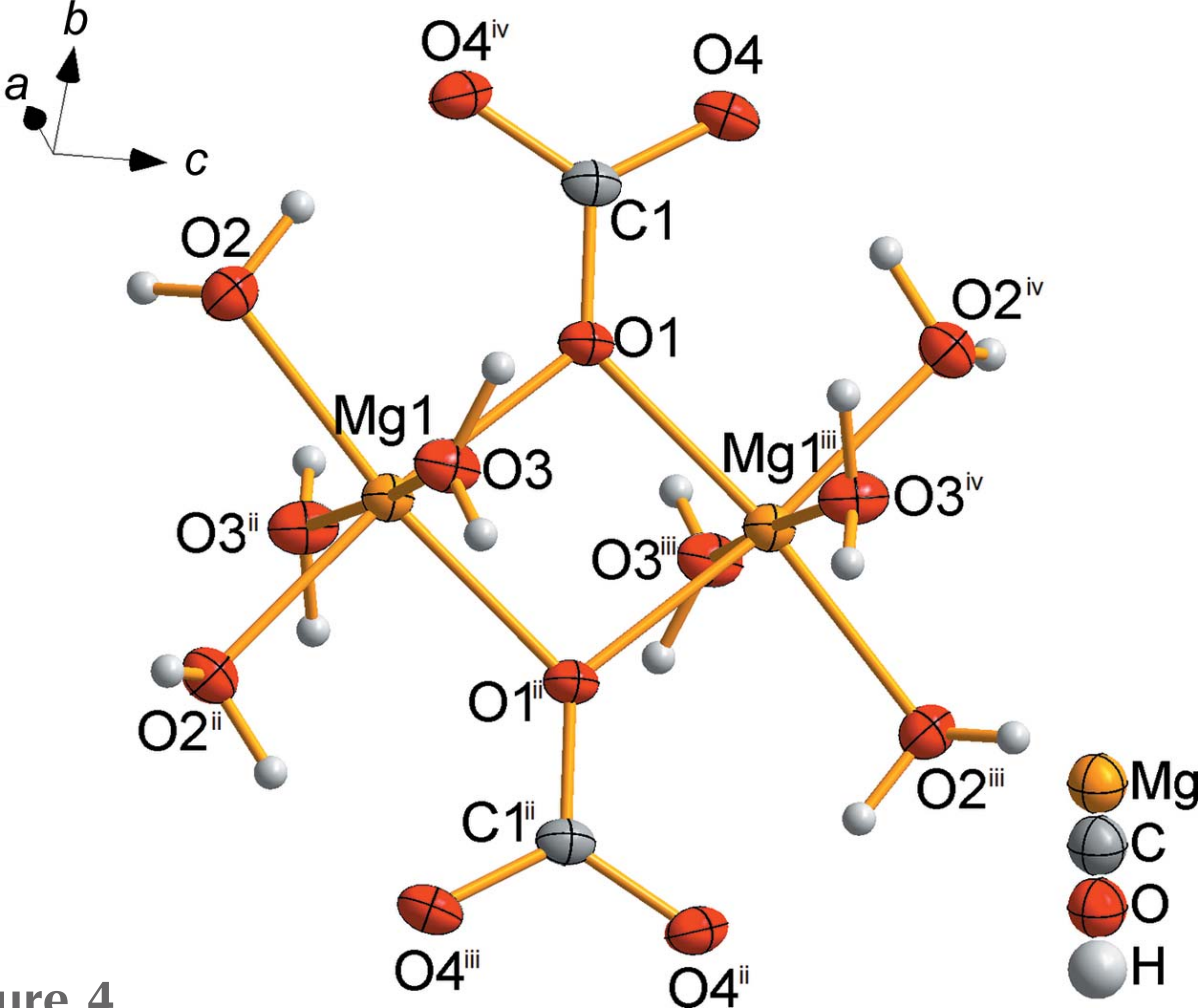
Illustration of the main building block in the crystal structure of MgCO_3_·6H_2_O, showing isolated pairs of edge-linked Mg(CO_3_)_2_(H_2_O)_4_ octa­hedra [symmetry codes: (ii) −*x* + 1, −*y*, *z*; (iii) −*x* + 1, −*y*, −*z* + 1; (iv) *x*, *y*, −*z* + 1].

Isolated pairs of edge-linked Mg(CO_3_)_2_(H_2_O)_4_ octa­hedra are the main building blocks in the crystal structure (Fig. 4 ▸). The crystal structure differs significantly from those of MgCO_3_·3H_2_O and MgCO_3_·5H_2_O; MgCO_3_·3H_2_O exhibits a monoclinic crystal structure consisting of infinite chains along [010], formed by corner-sharing MgO_6_ octa­hedra and CO_3_ groups, which link three MgO_6_ octa­hedra by two common corners and one edge (Giester *et al.*, 2000 ▸). In the monoclinic crystal structure of MgCO_3_·5H_2_O, the characteristic building units are isolated octa­hedra of [Mg(CO_3_)_2_(H_2_O)_4_]^2−^ and [Mg(H_2_O)_6_]^2+^ (Liu *et al.*, 1990 ▸).

The MgO octa­hedra in MgCO_3_·6H_2_O are slightly distorted (Table 2 ▸).

The carbonate units are linked in a monodentate manner to two magnesium ions across the O1 atom. They are planar and exhibit *C*_2*v*_ symmetry, because the C1—O1 bond is a little longer than the C1—O4 bond. Furthermore, the O1—C1—O4 angle is a little narrower than the O4—C1—O4^iv^ angle (see Table 2 ▸ for numerical data and symmetry code).

**Figure 5:**
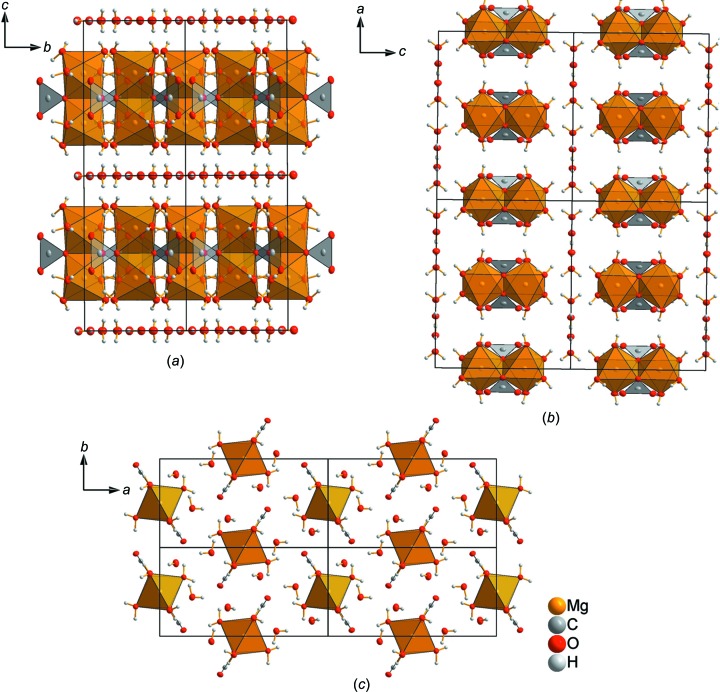
Projection of the crystal structure of MgCO_3_·6H_2_O (*a*) in the *a* direction, (*b*) in the *b* direction and (*c*) in the *c* direction.

**Figure 6:**
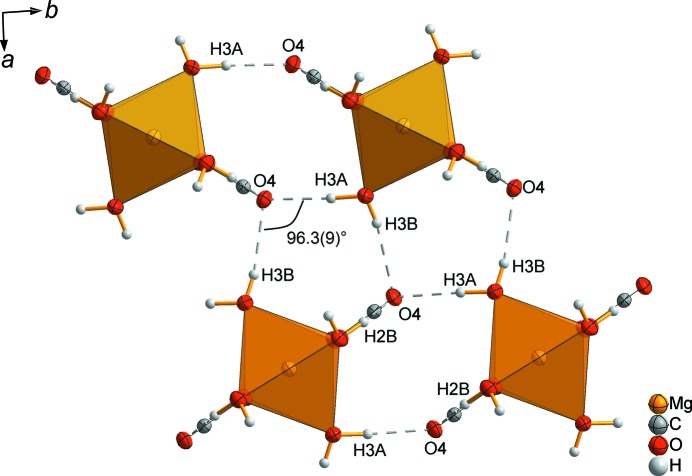
Part of the crystal structure of MgCO_3_·6H_2_O, showing the intra­layer hydrogen bonds (dashed lines).

The main building blocks are arranged in a sheet-like pattern, perpendicular to the *c* axis (Figs. 5 ▸ *a* and 5*b*). Within a sheet, every second main building unit is shifted along the [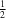, 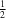, 0] direction and rotated by 90°. Consequently, a zigzag-like stacking order results (Fig. 5 ▸ *c*). The main building units in a sheet are linked by hydrogen bridging bonds (Fig. 6 ▸ and Table 3 ▸). The sheets are separated by layers of noncoordinating water mol­ecules in the (001) plane.

**Figure 7:**
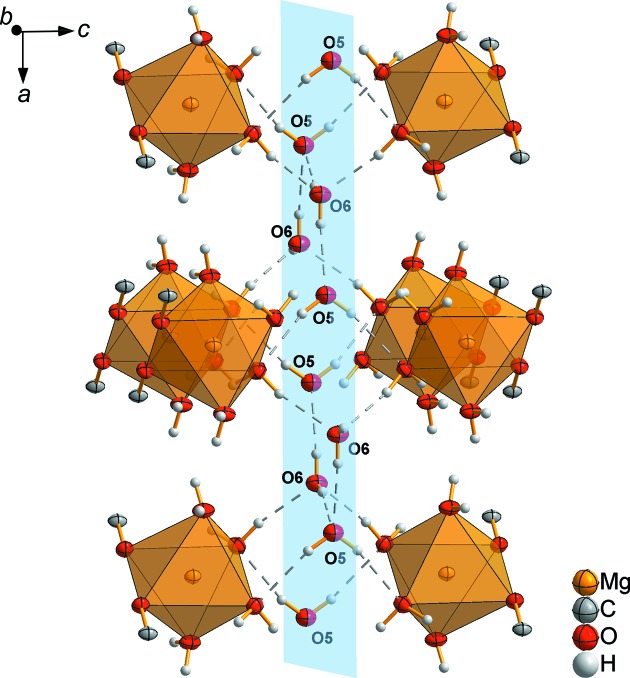
Part of the crystal structure of MgCO_3_·6H_2_O, showing the inter­layer hydrogen bonds (dashed lines). The (001) plane is shown in blue.

All the atoms of the noncoordinating H6*A*—O6—H6*B* mol­ecule are located in the (001) plane, whereas in the H5—O5—H5^i^ mol­ecule, only the O5 atom is situated in this plane (Fig. 7 ▸). The (001) plane is also the mirror plane of this mol­ecule. The noncoordinating water mol­ecules are linked by hydrogen bridging bonds both in the (001) plane among themselves and with the MgO octa­hedra. Thus, the crystal structure is three-dimensional crosslinked (Fig. 7 ▸).

### Comparison with crystal structures of other carbonate hydrates of bivalent metal ions   

Other neutral carbonate hydrates of bivalent metal ions with a water content greater than five units per formula are only known for calcium (CaCO_3_·6H_2_O) and nickel (NiCO_3_·5.5H_2_O). Like the title com­pound, these phases can be synthesized only at low temperatures of about 273.15 K and are transformed at room temperature to CaCO_3_ (Coleyshaw *et al.*, 2003 ▸) and amorphous nickel carbonate (Bette *et al.*, 2016 ▸; Rincke, 2018 ▸), respectively.

The crystal structure of CaCO_3_·6H_2_O is significantly different from that of MgCO_3_·6H_2_O for the very reason that the coordination number of the cation in CaCO_3_·6H_2_O is eight and not six as in MgCO_3_·6H_2_O (Dickens & Brown, 1970 ▸; Hesse *et al.*, 1983 ▸).

However, the radii of nickel and magnesium ions are very similar and actually the crystal structures of NiCO_3_·5.5H_2_O and MgCO_3_·6H_2_O exhibit similarities. Both crystal structures consist of isolated edge-linked pairs of *M*(CO_3_)(H_2_O)_4_ (*M* = Mg or Ni), which are the main building units and are arranged in sheets, together with noncoordinating water mol­ecules, perpendicular to the *c* axis. The symmetry of NiCO_3_·5.5H_2_O is lower; it crystallizes in the monoclinic group *P*2/*n*. As a consequence, there are two crystallographically different Ni atoms. In contrast to MgCO_3_·6H_2_O, in NiCO_3_·5.5H_2_O, the NiO octa­hedra of Ni2 are not rotated by 90° (Bette *et al.*, 2016 ▸).

## Conclusion   

A new neutral magnesium carbonate with the previously unknown high water content of six units per formula, *i.e.* MgCO_3_·6H_2_O, was produced by passing gaseous CO_2_ through an aqueous suspension of MgO and storing the filtered solution at 273.15 K. The X-ray diffraction pattern and Raman spectra confirmed the formation of the new phase and its transformation to MgCO_3_·3H_2_O. MgCO_3_·5H_2_O was not found in our study. Obviously, the formation conditions of magnesium carbonate hydrates depend on the concentration of the MgO suspension, the CO_2_ pressure, the temperature regime and the time of storage. Therefore, it would be useful to carry out further systematic investigations on the chemical kinetics of the formation of the magnesium carbonate hydrates.

The crystal structure of MgCO_3_·6H_2_O differs significantly from the other known magnesium carbonate hydrates, because the main building units are isolated pairs of edge-linked Mg(CO_3_)(H_2_O)_4_ octa­hedra and free water mol­ecules in the (001) plane, but it exhibits similarities to the nickel salt, NiCO_3_·5.5H_2_O (hellyerite).

## Supplementary Material

Crystal structure: contains datablock(s) I, global. DOI: 10.1107/S2053229620001540/lg3251sup1.cif

Structure factors: contains datablock(s) I. DOI: 10.1107/S2053229620001540/lg3251Isup2.hkl

Experimental conditions and caracterization. DOI: 10.1107/S2053229620001540/lg3251sup3.pdf

CCDC reference: 1981730

## Figures and Tables

**Table 1:** Experimental details

Crystal data	
Chemical formula	MgCO_3_·6H_2_O
*M*_r_	192.42
Crystal system, space group	Orthorhombic, *P* *b* *a* *m*
Temperature (K)	200
*a*, *b*, *c* (Å)	12.3564 (18), 6.5165 (7), 9.9337 (11)
*V* (Å^3^)	799.87 (17)
*Z*	4
Radiation type	Mo *K*α
μ (mm^−1^)	0.24
Crystal size (mm)	0.7 × 0.55 × 0.15

Data collection	
Diffractometer	Stoe IPDS 2T
Absorption correction	Integration (Coppens, 1970 ▸)
*T*_min_, *T*_max_	0.694, 0.887
No. of measured, independent and observed [*I* > 2σ(*I*)] reflections	8319, 1143, 965
*R*_int_	0.036
(sin θ/λ)_max_ (Å^−1^)	0.687

Refinement	
*R*[*F* ^2^ > 2σ(*F* ^2^)], *wR*(*F* ^2^), *S*	0.029, 0.085, 1.18
No. of reflections	1143
No. of parameters	83
H-atom treatment	All H-atom parameters refined
Δρ_max_, Δρ_min_ (e Å^−3^)	0.23, −0.26

Computer programs: *X-AREA* (Stoe & Cie, 2015 ▸), *X-RED* (Stoe & Cie, 2015 ▸), *SHELXS97* (Sheldrick, 2008 ▸), *SHELXL2016* (Sheldrick, 2015 ▸), *DIAMOND* (Brandenburg, 2017 ▸) and *publCIF* (Westrip, 2010 ▸).

**Table 2:** Selected geometric parameters (Å, °)

Mg1—O1	2.1043 (8)	C1—O4	1.2840 (11)
Mg1—O2	2.0859 (8)	C1—O1	1.2978 (17)
Mg1—O3	2.0672 (8)		
			
O3—Mg1—O2	86.12 (3)	O1—Mg1—O1^ii^	81.25 (5)
O2—Mg1—O2^i^	95.28 (5)	O4—C1—O4^iii^	120.41 (13)
O3—Mg1—O1	91.46 (4)	O4—C1—O1	119.79 (6)
O2—Mg1—O1	91.74 (3)	Mg1—O1—Mg1^ii^	98.75 (5)

Symmetry codes: (i) 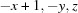; (ii) 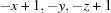; (iii) 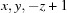.

**Table 3:** Hydrogen-bond geometry (Å, °)

*D*—H⋯*A*	*D*—H	H⋯*A*	*D*⋯*A*	*D*—H⋯*A*
O5—H5⋯O2^iii^	0.812 (18)	2.068 (19)	2.8545 (12)	162.9 (18)
O6—H6*B*⋯O5^ii^	0.87 (3)	1.93 (3)	2.7662 (19)	161 (2)
O6—H6*A*⋯O5^iv^	0.76 (3)	1.99 (3)	2.7137 (19)	160 (3)
O3—H3*B*⋯O4^v^	0.788 (19)	2.025 (19)	2.8055 (12)	170.7 (18)
O3—H3*A*⋯O4^vi^	0.93 (2)	1.77 (2)	2.7001 (12)	174.1 (18)
O2—H2*B*⋯O4^iii^	0.92 (2)	1.70 (2)	2.5868 (12)	162 (2)
O2—H2*A*⋯O6	0.84 (2)	1.91 (2)	2.7417 (13)	168.7 (18)

Symmetry codes: (ii) 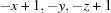; (iii) 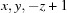; (iv) 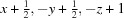; (v) 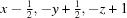; (vi) 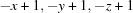.
