# Supplementary material for: A new hydrate of magnesium carbonate, MgCO3·6H2O
Source: Acta Crystallogr C Struct Chem. 2020 Feb 13;76(Pt 3):244–9. doi: 10.1107/S2053229620001540 (PMC7057182; doi:10.1107/S2053229620001540)
Supplement: Supplementary file 3 [file c-76-00244-sup3.pdf]

Supporting information

A new hydrate of magnesium carbonate: $MgCO_3 \cdot 6H_2O$

Christine Rincke,* Horst Schmidt and Wolfgang Voigt

Experimental conditions and characterization

Table S1 Conditions of the attempts for crystallization of hydrated magnesium carbonates.

Attempt	molalitiy(Mg^{2+}) in mol/kg(H_2O)	CO_2-pressure, duration of CO_2 discharge in hours	Temperature in K	Duration and conditions of storage	Product phases according to XRPD
V6	0,248	1 bar, 18 h	278.15	12 d, not stirred	$MgCO_3 \cdot 3H_2O$
V7	0,124	1 bar, 23 h	273.15	6 d, stirred	$MgCO_3 \cdot 3H_2O$
V8	0,124	1 bar, 22 h	273.15	2 d, stirred	$MgCO_3 \cdot 3H_2O$
V9	0,356	1 bar, 24 h	273.15	3 d, stirred	unknown phase[a)]
V10	0,388	1 bar, 24 h	273.15	1 d, stirred	unknown phase[a)]
V11	0,385	1 bar, 22 h	273.15	16 d, not stirred	$MgCO_3 \cdot 3H_2O$ + unknown phase[b)]

a) very fine crystals – too small for single crystal diffraction, b) crystals for suitable for single crystal diffraction.

Table S2 Comparison of band positions in Raman spectra of different magnesium carbonate hydrates.

$MgCO_3 \cdot 3H_2O$ (Coleyshaw *et al.*, 2003)	$MgCO_3 \cdot 5H_2O$ (Coleyshaw *et al.*, 2003)	$MgCO_3 \cdot 6H_2O$, present work	Assignment (Coleyshaw *et al.*, 2003)
2431, 2350	2628, 3264	3200	ν(OH)
	1705		δ(HOH)
1516, 1423	1514, 1424	1414	ν^{as}(CO)
1095	1098	1089	ν^{s}(CO)
713, 781	698, 774	710	δ^{as}(CO)
223	225	349, 299, 238, 190, 171, 151, 116	lattice vibrations

ν valence vibration, δ deformation vibration, s symmetric, as asymmetric
